# No role for electroencephalogram in the initial work-up of pediatric acute lymphoblastic leukemia

**DOI:** 10.1007/s00520-023-07692-9

**Published:** 2023-03-28

**Authors:** Anke Barnbrock, Natalia Lüsebrink, Susanne Schubert-Bast, Konrad Bochennek, Thomas Lehrnbecher

**Affiliations:** 1 Department of Pediatric Hematology and Oncology, Hospital for Children and Adolescents, Goethe University Frankfurt, Frankfurt, Germany; 2 Department of Neuropediatrics, Hospital for Children and Adolescents, Goethe University Frankfurt, Frankfurt, Germany; 3 Epilepsy Center Frankfurt Rhine-Main, Center of Neurology and Neurosurgery, Goethe University Frankfurt, Frankfurt, Germany; 4 LOEWE Center for Personalized and Translational Epilepsy Research (CePTER), Goethe University Frankfurt, Frankfurt, Germany

**Keywords:** Child, Acute lymphoblastic leukemia, Electroencephalogram, Diagnostics

## Abstract

**Purpose:**

The purpose of this study was to verify whether there is a prognostic benefit of electroencephalogram (EEG) performed during initial work-up of children with newly diagnosed acute lymphoblastic leukemia (ALL).

**Methods:**

In this retrospective monocenter study, we analyzed the value of electroencephalogram (EEG) performed during initial work-up of children with newly diagnosed acute lymphoblastic leukemia (ALL). All pediatric patients were included in this study who were diagnosed with de novo ALL in our institution between January 1, 2005, and December 31, 2018, and in whom an EEG was performed for initial work-up within 30 days of diagnosis of ALL. EEG findings were associated with the occurrence and the etiology of neurologic complications occurring during intensive chemotherapy.

**Results:**

Out of 242 children, EEG revealed pathological findings in 6 patients. Two of them developed a seizure at a later time point due to adverse effects of chemotherapy, whereas 4 children had an uneventful clinical course. In contrast, 18 patients with normal initial EEG findings developed seizures during therapy for different reasons.

**Conclusion:**

We conclude that routine EEG does not predict seizure susceptibility in children with newly diagnosed ALL and is unnecessary in the initial work-up as EEG investigation in young and often sick children requires sleep deprivation and/or sedation, and our data demonstrate no benefit in predicting neurological complications.

## Introduction

Acute lymphoblastic leukemia (ALL) is the most common childhood malignancy [1], with cure rates exceeding 90% [2]. It is standard of care that all children with newly diagnosed ALL undergo an initial work-up. These investigations are needed for staging, such as a lumbar puncture for detecting central nervous system (CNS) involvement, but also in order to prevent potential organ toxicity, such as echocardiography prior to the administration of anthracyclines. As it was recommended by a number of clinical trials, such as AIEOP-BFM ALL2009 (EudraCT 2007-004270-43), an electroencephalogram (EEG) was included in our institution in the initial work-up of children with newly diagnosed ALL in order to predict seizure susceptibility. As a clinical rational for this initial work-up is lacking, we aimed to critically assess in this study whether initial EEG investigation provides any additional information. This is important, as particularly in younger children, performing an EEG may need sedative medication and/or sleep deprivation.

## Patients and methods

All pediatric patients diagnosed with de novo ALL in our institution between January 1, 2005, and December 31, 2018, in whom an EEG was performed for initial work-up within 30 days of diagnosis, were included in the retrospective analysis. Exclusion criteria were start of therapy at another center, continuing therapy in a different treatment center, or presenting with severe neurological complications at the time of ALL diagnosis.

All patients were treated according to BFM-based protocols, such as AIEOP-BFM ALL2000, AIEOP—BFM ALL 2009, AIEOP-BFM ALL 2017 or Interfant 2006. Collected data included demographics and leukemia characteristics as well as the clinical course during treatment, in particular neurological complications. According to the clinical trials, CNS positivity was defined as >5 white blood cells/µl in the CSF and cytospin positive for blasts.

EEG was routinely performed during the initial work-up after diagnosis of ALL. EEG recordings were performed with scalp electrodes, based on international 10–20 electrode placement for school-aged children and reduced electrode number for younger children [3]. EEG diagnostics was repeated when patients presented during therapy with altered mental status, suspected seizure, or focal neurologic deficits. EEG results were described as normal, showing unspecific alteration (e.g., intermittent general background slowing) or pathologic (e.g., focal findings or interictal epileptiform discharges (IED), which were defined as focal, multifocal or generalized spikes, spike and waves, sharp waves, or sharp slow waves) [4, 5]. Mild alterations such as general background slowing are an expected phenomenon in severely ill children [6]. This general slowing may be accentuated by treatment with corticosteroids. In our analysis, we did only include pathologic findings, but not unspecific alterations. Children <5 years usually received sedative medication with melatonin, promethazine, or doxylamine succinate for sleep induction and/or sleep deprivation for EEG testing.

Written informed consent for medically indicated measures such as EEG and for data collection was obtained and documented within the consent procedures for cancer treatment that have been reviewed and approved by the local ethics committee (187-16).

## Results

A total of 242 patients (102 girls, median age 5.5 years (range 2 months to 24 years and 9 months)) were included in the analysis. One hundred ninety-eight patients (81.1%) were diagnosed with precursor B-ALL, 34 (14.0%) with T ALL, 4 (1.7%) with Philadelphia chromosome-positive ALL, 3 with B-ALL, and one patient each with t17/19 ALL, mixed phenotype, and undetermined leukemia, respectively. Ten patients (4.1%) were CNS-positive.

EEG findings at initial work-up were pathologic in 6 patients (2.5%) revealing IED (Table 1). All these patients were CNS negative. In three patients, cranial magnetic resonance imaging (MRI) was performed and did not reveal abnormalities. One patient suffered from autism; no neurological abnormalities were seen in the other 5 children. In none of these patients, antiseizure medication (ASM) was started. Whereas the neurological clinical course was uneventful in 4 patients, two patients developed symptomatic seizures: one patient during treatment with blinatumomab and one patient during re-induction, in whom MRI indicated posterior reversible encephalopathy syndrome (PRES). In both patients, ASM was started, and both recovered without developing epilepsy or long-term sequelae.

**Table 1:** Characteristics of patients with pathological EEG findings during initial work-up after newly diagnosed acute lymphoblastic leukemia

Age/sex	ALL subtype	CNS status	Initial cranial MRI	Initial EEG findings	Neurological findings	Anticonvulsive prophylaxis	Clinical course during ALL therapy	Etiology/time point of adverse event
2 y/f	pB ALL	Negative	n.d.	IED	Normal	None	Uneventful	-
3 y/m	pB ALL	Negative	n.d.	IED	Normal	None	Seizures	Blinatumomab (HR treatment)
15 y/m	pB ALL	Negative	Normal	IED	Normal	None	Uneventful	-
5 y/f	pB ALL	Negative	Normal	IED	Normal	None	Seizures	Chemotherapy (PRES); re-induction
3 y/m	pB ALL	Negative	n.d.	IED	Autism	None	Uneventful	-
6 y/m	pB ALL	Negative	Normal	IED	Normal	None	Uneventful	-

*y* year; *f* female; *m* male; *pB ALL* precursor B-ALL; *CNS* central nervous system; *MRI* magnetic resonance imaging; *n.d.* not done; *EEG* electroencephalogram; *PRES* posterior reversible encephalopathy syndrome; *IED* interictal epileptiform discharges

Normal EEG findings were seen in 197 (81.4%) patients at initial work-up, whereas 39 (16.1%) showed mild unspecific changes. Among these 236 patients without IED, 10 children (4.2%) were CNS-positive for ALL. In a total of 18 of the 236 patients (7.6%), neurological abnormalities were observed during therapy for ALL (one patient was CNS-positive for ALL) (Table 2). In 14 of the 18 patients without pathologic EEG findings, in whom a seizure occurred during ALL therapy, also a cranial MRI was performed during initial work-up which did not show any abnormality. Three patients each had clinical and radiological findings consistent with PRES, sinus venous thrombosis, or confirmed or suspected epilepsy, respectively. Two patients had an infectious episode, and three children had a seizure associated with medication (methotrexate (*n*=1) and anesthesia (*n*=2)). One patient had a seizure associated with encephalopathy of unclear origin. In three patients, the cause of seizures remained unclear.

**Table 2:** Characteristics of patients with normal initial EEG, who developed neurological abnormalities during treatment for acute lymphoblastic leukemia

Age/sex	ALL subtype	CNS status	Initial cranial MRI	Neurological complication during ALL therapy	Etiology/time point of adverse event
5 y/m	pB ALL	Negative	n.d.	Seizure	Unknown/re-induction
5 y/f	pB ALL	Negative	Normal	Seizure	Anesthesia/induction
8 y/m	T ALL	Negative	Normal	Seizure	Epilepsy/maintenance
16 y/m	T ALL	Negative	Normal	Seizure	Encephalopathy/induction
9 y/m	T ALL	Negative	Normal	Seizure	Chemotherapy (PRES)/induction
8 y/f	T ALL	Negative	Normal	Seizure	Chemotherapy (PRES)/induction
15 y/f	pB ALL	Negative	n.d.	Seizure	SVT/induction
2 y/f	pB ALL	Negative	Normal	Seizure	Fever/maintenance
1 y/m	pB ALL	Negative	Normal	Seizure	Anesthesia/induction
9 y/f	T ALL	Negative	Normal	Seizure	Suspected epilepsy/induction
5 y/m	pB ALL	Negative	Normal	Seizure	Suspected epilepsy/maintenance
12 y/m	pB ALL	Negative	Normal	Seizure	Chemotherapy (PRES)/induction
13 y/f	T ALL	Negative	Normal	Seizure	SVT/induction
6 y/m	pB ALL	Negative	Normal	Seizure	SVT/induction
3 y/f	pB ALL	Negative	n.d.	Seizure	Unknown/maintenance
4 y/m	pB ALL	Negative	n.d.	Seizure	Unknown/re-induction
11 y/m	pB ALL	Negative	Normal	Seizure	Methotrexate/consolidation
17 y/m	pB ALL	Negative	Normal	Seizure	Infection/re-induction

*y* year; *f* female; *m* male; *pB ALL* precursor B-ALL; *CNS* central nervous system; *MRI* magnetic resonance imaging; *n.d.* not done; *PRES* posterior reversible encephalopathy syndrome; *SVT* sinus venous thrombosis; the timing and triggering factors did not differ from those, who had a seizure following an unremarkable staging EEG report

## Discussion

Our data show that neurological abnormalities, in particular seizures, are not uncommon during treatment of children with ALL [7, 8] and that patients do not benefit from an EEG performed during initial work-up. In our retrospective analysis, EEG revealed pathologic findings in 6 patients (2.5%). Although none of these children received pre-emptive ASM, 4 of them had an uneventful clinical course. In contrast, 2 children developed seizures at a later time point, both due to well-described adverse effects of therapy. Importantly, the frequency of IED detected during initial work-up in our patient population matches the reported frequency of 3% in healthy children [9]. Whether pre-emptive anticonvulsive treatment in patients with ALL and pathologic EEG group would have been beneficial remains unclear.

Studies from the 1990s showed that up to 60% of pediatric patients with ALL already have alterations in the EEG at the time of diagnosis [10–12]. Our analysis of EEG investigation during initial work-up revealed normal or unspecific alterations in 236 patients (97.5%). This population includes 39 patients (16.1%) with mild general abnormalities such as non-epileptic intermittent general background slowing, which might be attributed to the reduced general condition of the patient as well as to diagnostic and therapeutic interventions such as sedation for lumbar puncture or methotrexate administration into the CSF. The fact that we did not include unspecific EEG findings in our analysis could explain, at least in part, the differences between our results and previous studies, but it can also be speculated whether different technical equipment and different handling of sedating medications in the context of EEG recording could have also contributed to these discrepancies. A total of 18 patients (7.4%) with non-pathologic initial EEG developed symptomatic seizures. This observation is in line with previous studies reporting that approximately 5–10% of children with ALL develop a seizure during treatment [13, 8]. The majority of these events occurred early in the course of therapy and could be attributed to well-known triggers such as sinus venous thrombosis or cytotoxic agents, e.g., methotrexate [14, 7]. It is noteworthy that all EEGs were performed as part of the routine work-up prior to any neurological abnormality including seizure. In fact, there was only one patient who presented with severe neurological symptoms due to resuscitation prior to diagnosis of ALL and was therefore excluded from our analysis. The patient group experiencing a seizure during induction had their routine EEG within the first 3 days after diagnosis. Several children developed seizures late during therapy, which were mostly attributable to causes such as high fever, and thus were most likely unrelated to therapy of leukemia. This hypothesis is supported by the observation that the prevalence of seizures at a later stage of therapy is comparable to that of the age-matched general population [15]. Previous analyses have even demonstrated that screening EEGs after completion of ALL treatment did not have a prognostic benefit in detecting neurological sequelae [16].

Recently, we have reported that routine MRI examination as initial work-up does not add any information regarding treatment of children with ALL nor does it improve the detection of CNS involvement compared with CSF analysis alone. These data resulted in a change of our internal standard [17]. Similarly, our present data demonstrate that EEG findings during initial work-up are not helpful in predicting neurological complications during therapy for ALL. Interestingly, all pathologic EEG findings were observed in children without CNS involvement, and all but one neurological complication occurred in patients in whom CSF was free of leukemia blasts.

In conclusion, as EEG investigation in young and often sick children diagnosed with ALL requires sleep deprivation and/or sedation, and as our data demonstrate no benefit in predicting neurological complications, we omitted EEG as routine investigation during initial work-up in children with ALL.

## Data Availability

All data and material related to this study are available on request.

## Abbreviations

ALL: Acute lymphoblastic leukemia
CNS: Central nervous system
EEG: Electroencephalogram
CSF: Cerebrospinal fluid
MRI: Magnetic resonance imaging
IED: Interictal epileptiform discharges
ASM: Antiseizure medication
PRES: Posterior reversible encephalopathy syndrome
